# The Influence of Structure Heights and Opening Angles of Micro- and Nanocones on the Macroscopic Surface Wetting Properties

**DOI:** 10.1038/srep21400

**Published:** 2016-02-19

**Authors:** Ling Schneider, Milan Laustsen, Nikolaj Mandsberg, Rafael Taboryski

**Affiliations:** 1 Department of Micro- and Nanotechnology, Technical University of Denmark, 2800 Kongens Lyngby, Denmark

## Abstract

We discuss the influence of surface structure, namely the height and opening angles of nano- and microcones on the surface wettability. We show experimental evidence that the opening angle of the cones is the critical parameter on sample superhydrophobicity, namely static contact angles and roll-off angles. The textured surfaces are fabricated on silicon wafers by using a simple one-step method of reactive ion etching at different processing time and gas flow rates. By using hydrophobic coating or hydrophilic surface treatment, we are able to switch the surface wettability from superhydrophilic to superhydrophobic without altering surface structures. In addition, we show examples of polymer replicas (polypropylene and poly(methyl methacrylate) with different wettability, fabricated by injection moulding using templates of the silicon cone-structures.

Tuning surface wettability by engineering surface structure and/or surface chemistry has attracted huge research interests, due to the promising applications of these surfaces in areas of self-cleaning, anti-icing, drag reduction, anti-fogging and microfluidic devices^1^,^2^,^3^,^4^,^5^,^6^,^7^,^8^,^9^,^10^,^11^,^12^. A general approach is to texture low surface energy materials to obtain superhydrophobic surfaces, and high surface energy materials to obtain superhydrophilic surfaces. These artificial structures are typically inspired by nature, for example, by lotus leaves^13^, water strider’s legs^14^, moth eyes^3^ and etc^15^,^16^. In addition to creating biomimetic structures, there are also studies, where periodic artificial structures of different geometry are fabricated for obtaining a better theoretical understanding of the wetting phenomena to benefit the future structural design^17^,^18^,^19^,^20^,^21^. Though there have been a few studies on the influence of height, width, and period of nano- and micro-cones on the surface wettability^9^,^22^, the influence of the structural include/opening angles on the surface wettability has rarely been discussed. The fabrication of tapered cones require some predesigned templates^2^,^23^,^24^, pre-moulding methods^25^, or fine adjustment of processing parameters^26^,^27^, which makes it difficult to tune the opening angles of the cones. However, the cone geometry is usually closer to the aforementioned natural structures than the flat pillars or holes; and it has been suggested that the opening angle of the cones can have crucial influence on the sample wettability^9^,^28^.

Reactive ion etching (RIE) is a dry etching technique that can be used to structure silicon surfaces without masks through the combined effect of a corrosive gas (SF_6_ and/or CH_4_) and a passivating gas (O_2_)^26^,^29^,^30^,^31^,^32^. As most of the surfaces made this way appear black due to the scattering of the incident light by nano- and submicron structures, this method is also known as the “black silicon method”, and has widely been used in optical applications such as anti-reflective surfaces^26^,^31^,^32^. By tuning the processing parameters, namely gas flow rate, platen power, chamber pressure, and etching time, it is possible to alter the structure shape, and even the opening angles of the cones^9^. However, to the best of our knowledge, there are not yet reports on applying this method to systematically study the influence of structural opening angle on the surface wettability.

In this paper, we report a systematic study of surface wettability versus structural opening angles of the cones. We fabricate the nano- and microcones (structure heights range from less than 100 nm to around 2 μm) using RIE and alter the structural geometry by varying the processing time and gas flow rates. By using hydrophobic coating or hydrophilic surface treatment, we are able to switch the surface wettability from superhydrophilic to superhydrophobic without altering surface structures. We will discuss the influence of structure height and opening angle on sample superhydrophobicity, measured as the static contact angle and the roll-off angle. In addition, we will show some examples of polymer surfaces (polypropylene, PP and poly(methyl methacrylate), PMMA) with different wettability. Our technique will pave the way for the design and fabrication of large scale and low cost functional surfaces.

## Results and Discussion

In this study, we altered the flow rate of SF_6_ and O_2_ and the etching time 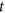, and kept the rest of the parameters unchanged, as the gas flow rate is the governing parameter for tuning the opening angles of the resulting cones^26^,^31^,^32^. As the absorbance of light varies with the geometric features of the structures, such as, height, aspect ratio, and period of the structures^26^,^31^,^32^,^33^,^34^, our samples do not necessarily appear black; some are black and some appear brownish.

### Superhydrophobic silicon samples

**Figure 1:**
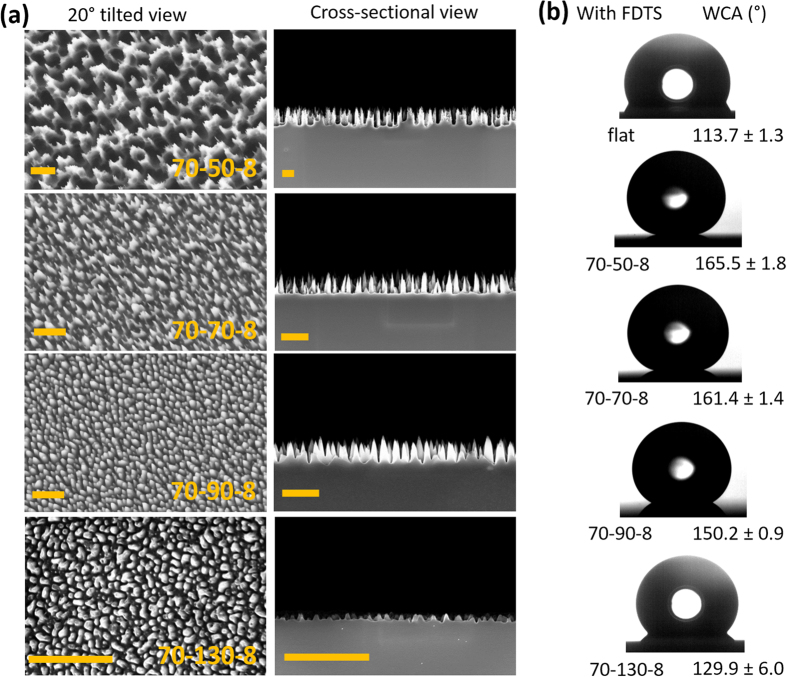
(**a**) Representative scanning electron microscopy images (20° tilted view and cross-sectional view) of nano- and submicron cones prepared with different flow rates of SF_6_ and O_2_ gases. The flow rates of SF_6_ and O_2_ vary from 70–50, 70–70, 70–90, to 70–130 sccm. For simplicity, we denote samples of different SF_6_ and O_2_ flow rates and etching time as 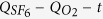. Therefore 70–50–8 means a sample processed with 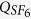 = 70 sccm, and 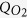 = 50 sccm for 8 min. The detailed processing information can be found in the supporting information (SI). Scale bars are 1 μm. (**b**) Images of sessile water droplets on flat and structured Si surfaces with FDTS coating, and the corresponding measured static contact angles. Droplet volume is 10 μl.

**Figure 2:**
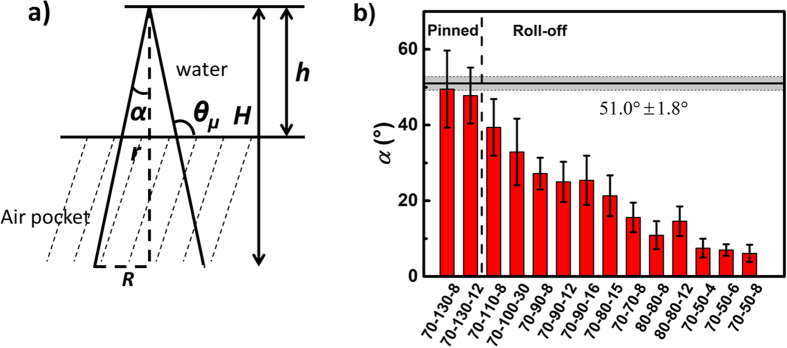
(**a**) Side view of a partially wetted cone; (**b**) column graph of samples of different opening angles fabricated by different processing parameters (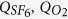 and *t*). The detailed processing parameters, and the corresponding heights, opening angles, and contact angles can be found in Table S1.

Figure 1a shows scanning electron microscopy (SEM) images of Si surfaces prepared by RIE for 8 min, using SF_6_ of 70 sccm and O_2_ of various flow rates: 50, 70, 90, and 130 sccm respectively. For simplicity, we denote samples of different SF_6_ and O_2_ flow rates and etching time as 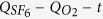. Therefore 70–50–8 means a sample processed with 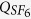 = 70 sccm, and 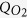 = 50 sccm for 8 min. Morphologies of samples vary from sharp hierarchical needles (70–50–8), tapered cones (70–70–8 and 70–90–8), to rounded domains (70–130–8), which are macroscopically uniform while microscopically stochastic. According to the cross-sectional SEM images, structures prepared with different O_2_ flow rates are of different heights: with increasing O_2_ rate, the structure height reduces from 1.540 ± 0.544, 0.733 ± 0.079, 0.557 ± 0.084, to 0.264 ± 0.026 μm. This trend is consistent with results obtained by other groups^26^,^31^,^32^. As the height of the fabricated structures ranges from a few hundred nanometers to a few micrometres, we refer to our structures as nano- and microcones. Results of static water contact angle measurements of these surfaces in comparison to the flat Si surface are shown in Fig. 1b. For samples with the hydrophobic 1 H, 1 H, 2 H, 2 H-perflourodecyltrichlorosilane (FDTS) coating, with increasing O_2_ flow rate, we measured decreasing static water contact angle. By assuming that most samples exhibit a (tapered) cone shape, the geometry of these samples is determined by only two parameters, the height and the opening angle 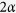 of the cones (Fig. 2a). It is thus necessary to discuss which geometric parameters dominate the wetting properties of a chemically fixed surface.

As depicted in Fig. 2a, the water-air interface is almost flat on the microscopic scale at a quasi-equilibrium^9^,^28^. The micro-contact angle at the water-solid sidewall 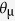 (Fig. 2a) is thus determined by the advancing contact angle 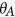 of the flat FDTS surface. This is to say, for the non-wetting condition, the Cassie-Baxter state^35^, or an intermediate Cassie-impregnating state^28^,^36^, 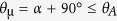; while for the complete wetting situation, the Wenzel state^37^, 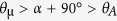. Hence for the non-wetting condition, the opening angle of an individual cone has to fulfil 2*α* ≤ 51.0° ± 1.8°. Figure 2b summarizes the opening angles of 14 samples and their corresponding wetting states. Among all 14 measured samples, two are sticky, that is water droplets were pinned to the surface even when the surface was positioned upside down; and the rest are non-sticky, that is water droplets rolled/slid off from the surfaces at a certain surface inclination. Apparently, all the non-sticky samples have opening angles smaller than the threshold value 51.0° ± 1.8°. The two sticky samples have however opening angles of 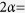 49.5° ± 10.2° (70–130–8) and 2*α* = 47.8° ± 7.4° (70–130–12), which are above or comparable to the expected threshold. Interestingly, for Si-70–130–8, all 5 tested drops were pinned; while for Si-70–130–12, only 3 out of 5 drops were pinned, which fits very well with this threshold hypothesis. It is worth mentioning that here we use the critical angle to draw a distinct line between the slippery and sticky surfaces. However, the degree of slipperiness and stickiness is always gradual. In fact, the wetting test on sample Si-70–130–12 shows that depending on structural imperfections on the microscale, the surface can be either “slippery” and “sticky” on macroscale.

**Figure 3:**
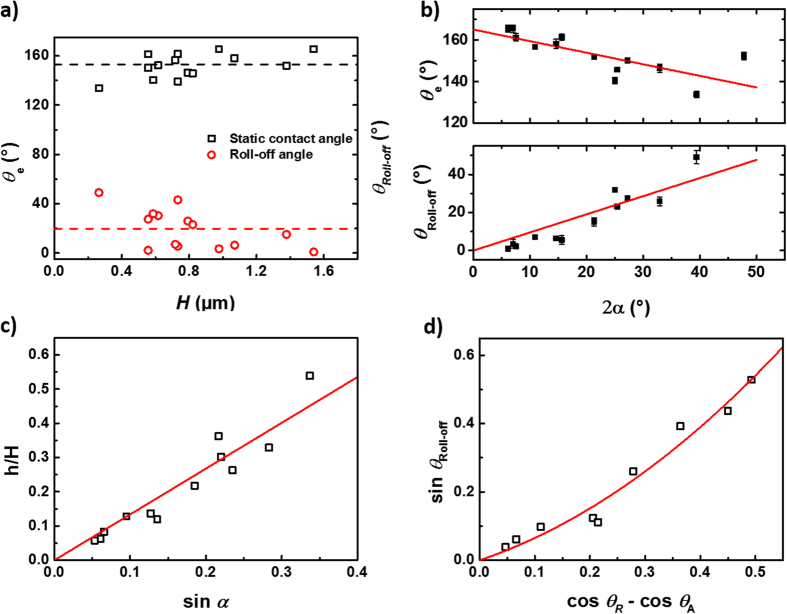
Dependence of static contact angles (black squares) and roll-off angles (red circles) on *H* (**a**) and 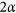 (**b**) of nano- and submicron cones; (**c**) dependence of water penetration degree on opening angle, *h*/*H* vs sin α; (**d**) dependence of the roll-off angle on the contact angle hysteresis, 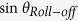 vs 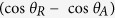. The dash lines serve as guides to the eye, while the solid lines are linear (**b,c**) and polynomial (**d**) fits of the experimental data.

Though results from Fig. 2b fit the critical opening angle assumption without considering the influence of the structural height, the influence of structural height on the wetting properties of individual samples should not be excluded completely. To understand the influence of the opening angles and structure heights, we plot water contact angle and roll-off angle of different samples with respect to both 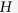
and 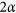 (Fig. 3). Interestingly, the static and dynamic contact angles do not show obvious dependence on 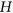 but exhibit an apparent linear dependence on 2*α* for the cones.

The apparent contact angle at equilibrium 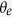 of a rough surface is usually described by the Cassie-Baxter equation for the non-wetting state^9^,^35^


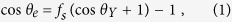


where 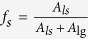, is the solid fraction.

For a cone geometry depicted in Fig. 2a,


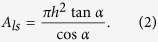



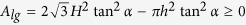
, for hexagonal close-packed cones. Equation 1 can be rewritten as,


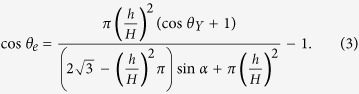


According to the Cassie-Baxter equation (Eq. 1), the larger the solid fraction 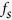, the smaller the 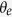, as both 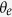 and the Young contact angle 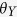 are larger than 90°. The *θ*_*e*_ ~ 2*α* dependence from Fig. 3b can only be achieved if *h*/*H* increases with increasing 2*α*, indicating that the penetration depth 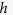
depends on 2*α* for the given geometry. Such dependence is consistent with our data analysis, as shown in Fig. 3c, where the water penetration degree *h*/*H* is shown to depend on 2*α* linearly. Here *h*/*H* is calculated by Equation 3, using the experimentally measured angles 2*α* and 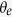, and ranges from 5% up to 50%.

The wetting situations described by the Cassie and Wenzel models are usually considered binary; there is no gradual transition from Cassie to Wenzel state (or vice versa); a surface should be either non-wetted or completely wetted. However, several studies have revealed that an intermediate wetting state exists, which can be reversed back to the Cassie state when certain conditions are met^16^,^28^,^36^,^38^. Although we here use the Cassie-Baxter model to explain the linear dependence of 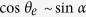, it does not necessarily mean that the intermediate state does not exist for some of the geometries presented.

We now turn to describe the dynamic properties of the surfaces. The linear increase of roll-off angle with increasing opening angle shown in Fig. 3b also indicates that the structures are partially wetted with various water penetration degrees. Such a wetting situation can be understood in two ways: 1. The structures are not perfect – some are sharper and others are blunter. Therefore, there is a stochastic mixture of totally non-wetted and wetted cones on the microscale, which results in the partial wetting situation on macroscale. 2. In an ideal situation, all cones are identical and arranged in hexagonal close packed order. The cones are wetted exactly the same way on the microscale with certain degree of water penetration. The wetting situation of structures fabricated by RIE mostly falls into the first category due to the nature of the RIE technique^29^. The macroscopic wetting behaviour is none-the-less the same for both cases.

The roll-off angle 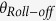 is determined by the balance of the driving force along the rolling plane and the friction during the movement, which is also called the pinning force, and is proportional to the contact angle hysteresis and the surface tension of the liquid. For a flat surface, the relation between a roll-off angle and the contact angle hysteresis can be explained by the Furmidge equation^39^.


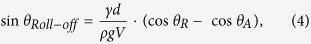


where 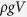 is the gravity force acting on the droplet, which is constant for all measured droplets, *d* is the width of the drop base viewed along the rolling/sliding direction, and 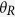 and 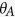 are the receding and advancing contact angles respectively. For rough surfaces, instead of using the width *d* of the drop, the effective three-phase contact length 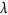 along the rolling direction should be used^18^,^21^. For the hexagonal closed packed cone model geometry, this length is given by 
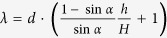
. The Furmidge equation can thus be rewritten as:


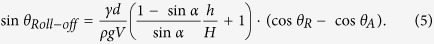


When we plot the roll-off angle versus contact angle hysteresis (Fig. 3d), we are able to fit the plot with a second order polynomial fit. The second order dependence implies, for our structures, that 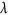 has a linear dependence on the contact angle hysteresis. Such dependence is supported by the plot of 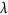 versus contact angle hysteresis (Figure S3).

It is worth mentioning that Equations 3 and 5 are derived based on the hexagonal closed packed cone geometry, which is the most representative geometry of the fabricated nano and microcones. However, not all samples exhibit the closed packed geometry, as can be seen from both Fig. 1 and Fig. S1. In the supporting information (SI), we discuss several other packing geometries, which nonetheless only change some prefactors in Equations 3 and 5.

### Superhydrophobic polymers

**Figure 4:**
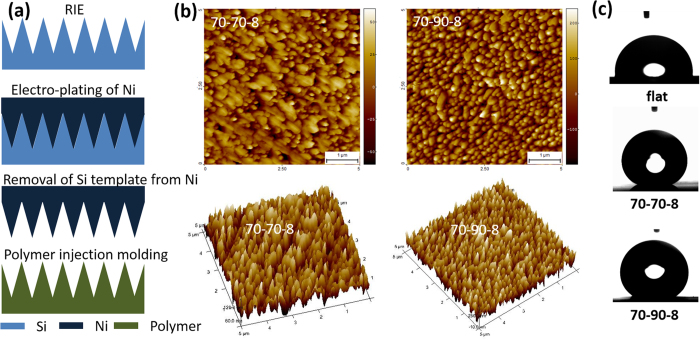
(**a**) schematic steps of fabrication of polymer replicas; (**b**) 2D and 3D AFM images of PP replicas fabricated via two different silicon masters 70–70–8 (*H* ~ 120 nm), and 70–90–8 (*H* ~ 460 nm); (**c**) Images of water droplets on flat and structured PP surfaces.

Though much effort has been invested to study the wetting properties of silicon samples, the final goal is to develop a technique that can be integrated into existing fabrication techniques for solid materials, such as injection moulding of polymers. As a starting point and a simple demonstration of our technique, we chose structures 70–70–8 and 70–90–8 for master origination and a low surface energy material polypropylene (PP, γ_*PP*_ ≈ 29 mN/m^40^) for injection moulding. Though Si-70–50–5 exhibits the strongest superhydrophobicity, replication fidelity of this structure could be low due to its hierarchical nature; while 70–70–8 and 70–90–8 with FDTS coating still have high superhydrophobicity and are easy to replicate. The main steps for replicating polymer surfaces through Si masters are shown in Fig. 4a. The prepared Si master serves as a template for forming textured Ni shim, and is removed by warm KOH bath after the Ni formation (supporting information). The Ni shim is then used as a mould for injection-moulding textured PP samples. A more detailed fabrication procedure can be found in the supporting information (SI) and our previous publications^28^,^41^.

Both replicated PP samples exhibit cone morphology after injection moulding (Fig. 4b), indicating that the structures have been transferred from their Si masters successfully. However, the cones of PP-70–70–8 are much shorter than those of PP-70–90–8, though their masters are quite the opposite: the cones of Si-70–70–8 are 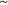
170 nm higher than those of Si-70–90–8. The replication quality of injection moulded polymer samples depends mainly on the filling quality of the polymer melt and the demoulding of the structures from the mould. The filling of micro-cavities in Ni-70–70–8 is more difficult than in Ni-70–90–8, as the former has a higher aspect ratio (Si-70–70–8 around 3.6 and Si-70–90–8 around 2.1)^42^,^43^. On the other hand, the higher surface area of structure 70–70–8 results in a higher friction during the demoulding, which might cause deformation or even fracture of the moulded polymer cones^44^. Interestingly, the water wettability of the two samples however follows the trend of their silicon masters: PP-70–70–8 is more superhydrophobic than PP-70–90–8, as summarized in Table 1. As discussed previously, the structure height contribute less to the superhydrophobicity compared to the structure opening angle or the structure aspect ratio, which explains why samples with shorter cones exhibit higher superhydrophobicity.

For samples of the same surface morphology, their superhydrophobicity is mainly dominated by the surface chemistry. As FDTS has a lower surface energy than PP, the resulting Si surface coated with FDTS should be more superhydrophobic than the PP surface, if we assume both have the same surface structure. The measured water contact angles of Si-70–70–8 and PP-70–70–8 follow such a rule, i.e. Si-70–70–8 is slightly more superhydrophobic than PP-70–70–8. However, PP-70–90–8 is more superhydrophobic than its silicon master Si-70–90–8. Such a deviation from the theory could possibly be due to the fact that the structure of PP-70–90–8 is different than its Si master Si-70–90–8. During demoulding, the PP cones might be stretched, which might form sharper cones than their Si masters.

### Superhydrophilic silicon samples

**Figure 5:**
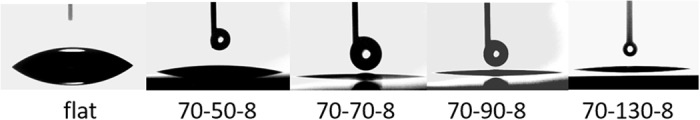
Images of droplets on uncoated silicon surfaces processed at different etching parameters: 70–50–8, 70–70–8, 70–90–8, and 70–130–8. The photos were taken while the droplet was still impacting the surface, so the water contact angle measured here should be the advancing contact angle.

The complete wetting can be described by the Wenzel equation^37^


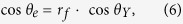


with 
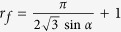
 being the surface roughness factor for the cone shape model, which equals the surface area divided by the apparent area of the rough surface. For *θ*_*Y*_ < 90°, creating surface roughness would lead to more hydrophilic surfaces, and even superhydrophilic surfaces, according to the Wenzel model. The water contact angle of unstructured Si surface after plasma cleaning is 35.5 ± 4.1°, and 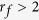 for all samples, as calculated from the opening angle 2α measured on the SEM images. The resulting 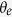 should thus be much smaller than 10° for the structured surfaces. We therefore tested the wetting properties of samples processed by the same SF_6_ and O_2_ flow rates as the ones shown in Fig. 1a, but without FDTS coating (Fig. 5). All surfaces exhibited strong superhydrophilicity during the measurements: the water droplet spread on the surface immediately after impacting the surface. The static contact angles of all samples are smaller than 5 degree. Interestingly, according to the Wenzel equation, for complete wetting, the higher the surface roughness, the smaller is the contact angle if *θ*_*Y*_ < 90°. However, here we find the highest contact angle ∼10° for the surface with the highest 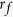. The high water contact angle of 70–50–8 is perhaps the result of the hierarchical nature of this sample surface (Fig. 1a). Hejazi *et al.* suggested that for hierarchical surfaces there could be two wetting states at different levels of roughness; structures that contribute to the first order roughness, the small spikes on top of the cones are completely wetted, while structures that contribute to the second order roughness, the cones might be partially wetted (air trapped)^45^. Such a multi-degree wetting situation can result in a higher apparent contact angle 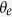, as demonstrated by Hejazi *et al.*, which is consistent with our experimental observation on superhydrophilic Si-70–50–8.

### Hydrophilic polymer replicas

For a simple demonstration of our method, we textured PMMA to generate the hydrophilic function, as the surface energy of PMMA (*γ*_*PMMA*_ ≈ 39 mN/m^40^) is relatively high among polymers and can be injection-moulded. To further improve the wettability of the textured surface, we treated the textured surface with O_2_ plasma for 25 s. As proved by AFM results (Figure S6), there is no obvious surface structure modification within such a short treatment.

**Figure 6:**
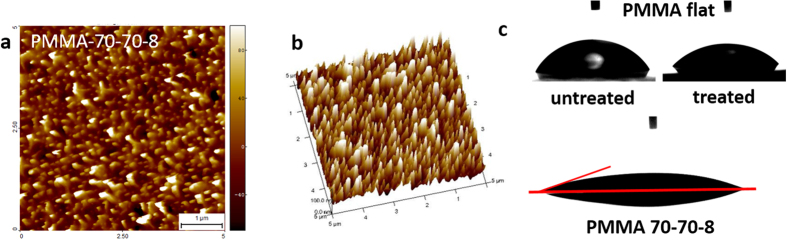
2D (**a**) and 3D (**b**) AFM images of treated PMMA replica fabricated via silicon masters 70–70–8 (H~120 nm); (**c**) Photos of water droplets on flat (with and without plasma treatment) and textured PMMA (plasma treated) surfaces.

Figure 6 represents PMMA samples fabricated through the silicon master 70–70–8, which is the same one used for fabricating PP samples. Here the height of the cones is only around 120 nm, which is similar to that of PP–70–70–8, but also much smaller than that of the silicon master Si–70-70–8. Such a small height is probably due to the same reason: the polymer melt did not fully fill the Ni cavities, and/or the cones broke during the demolding. The Ni insert used for injection moulding was examined by SEM afterwards, however no obvious residual polymer was found in the holes (Figure S7). We therefore attribute the low structure height to the incomplete filling of the submicron cavities. Though the structures are a bit lower than expected, the surface wettability has increased after texturing: The water contact angle is 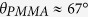 for flat untreated PMMA, 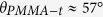 for treated PMMA and 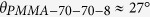 for treated and textured PMMA. We can thus conclude that by texturing and surface treating PMMA, we can mass–produce surfaces with high water wettability.

In summary, we have discussed the influencing geometric parameters on surface wettability at a given surface chemistry. From our experimental results, the opening angles of the nano- and microcones play a dominant role on both the static contact angle and the roll-off angle of a surface: the static and dynamic contact angles of superhydrophobic silicon samples exhibit linear dependence on the opening angles of the cones, whereas there is no obvious dependence of them on the height of the cones. Such a rule also applies to polymer samples, injection moulded using the corresponding silicon masters. In addition, we have demonstrated that the same structures used for superhydrophobic applications also can be used to fabricate superhydrophilic surfaces, without any further surface modifications required. The multi-order roughness of the hierarchical surface, however, reduces the superhydrophilicity slightly, due to the different wetting states of the various levels of roughness.

## Methods

### Fabrication of superhydrophobic and superhydrophilic silicon samples

Silicon wafers (100) with nano- and microcones were fabricated using reactive ion etching (RIE, Pegasus D-RIE, STS, UK). By tuning SF_6_ and O_2_ flow rate and etching time during the etching, nano- and microcones of different heights and aspect ratios were fabricated. Detailed fabrication parameters can be found in the supporting information (SI). After RIE, all samples were cleaned by N_2_/O_2_ plasma (N_2_ 400 sccm, O_2_ 70 sccm, power 1000 W) for 30 min. For superhydrophobic applications, an additional monolayer of 1 H, 1 H, 2 H, 2 H-perflourodecyltrichlorosilane (FDTS) was deposited by molecular vapor deposition (MVD, MVD 100, Applied Microstructures, USA) immediately after the plasma treatment, using a standard recipe. The detailed parameters can be found in the supporting information (SI). While for superhydrophilic applications, the samples were used directly after the plasma treatment.

### Fabrication of nickel mold

Nickel vanadium (NiV) of 100 nm was sputter-coated (CMS-18, Kurt J. Lesker Company®, USA) onto the Si master wafers to serve as a seed layer. A nickel (Ni) layer of 300 μm was deposited via electroforming. The Si template was then dissolved in a warm KOH bath (30 wt%, 80 °C). A monolayer of 1 H, 1 H, 2 H, 2 H-perflourodecyltrichlorosilane (FDTS) was coated by molecular vapor deposition (MVD, MVD 100, Applied Microstructures, USA) onto the resulting Ni shim. This layer was used as an anti-sticking layer for removing the polymer parts from the mold. Fabrications of both Si masters and Ni molds were done in the cleanroom (class 10–100).

### Injection molding of polymer samples

Polymer samples were injection molded using a variotherm heating system, where the mold temperature is first heated to a higher temperature for maximal filling and cooled down to a lower temperature for easy demolding.

### Contact angle measurements

Contact angles were measured using an optical tensiometer (Theta, Attension, Finland) with a high-speed camera (3000 fps, MotionXtra N3, IDT, USA) and tilted cradle. Shapes of droplets were fitted by polynomial fitting for both static and dynamic contact angle measurements. For all contact angle measurements presented in the main text, droplets of 10 μL in volume were used. To ensure that the measured static contact angles do not depend on the drop volume, we compared the measurements on same samples obtained with droplets of both 5 and 10 μl in volume (Figure S2). The deviations of the static contact angles are within the error range. The baseline was determined manually. For each sample, five measurements were made at different locations on the surface.

### Characterization of nano- and microcones

Structured Si samples were characterized by scanning electron microscopy (SEM, Supra 40 VP, Carl Zeiss AG, Germany) both at cross-sectional view and at 20° tilted view. The heights of the nano- and microcones were determined using software provided by Zeiss and the opening angles were manually measured by Image J. The detailed description and the discussion regarding the accuracy of the measurement can be found in the supporting information (SI). Polymer samples were imaged by atomic force microscopy (AFM, Dimension Icon, Bruker Corporation, USA).

## Additional Information

**How to cite this article**: Schneider, L. *et al.* The Influence of Structure Heights and Opening Angles of Micro- and Nanocones on the Macroscopic Surface Wetting Properties. *Sci. Rep.* **6**, 21400; doi: 10.1038/srep21400 (2016).

## Supplementary Material

Supplementary Information

## Figures and Tables

**Table 1:** Static contact angles and roll–off angles of PP replicas and their corresponding Si masters.

	Si 70–70–8	PP 70–70–8	Si 70–90–8	PP 70–90–8
Static contact angle (°)	161.4 ± 1.4	157.7 ± 2.8	150.2 ± 0.9	150.8 ± 1.6
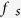 (%)	8.7	8.2	22.1	13.9
roll–off angle (°)	5.6 ± 2.2	13.5 ± 1.6	27.5 ± 0.9	24.8 ± 2.4

*f*_s_ was calculated from the Cassie equation (Eq. 3).
