# Supplementary material for: The Influence of Structure Heights and Opening Angles of Micro- and Nanocones on the Macroscopic Surface Wetting Properties
Source: Sci Rep. 2016 Feb 19;6:21400. doi: 10.1038/srep21400 (PMC4759530; doi:10.1038/srep21400)
Supplement: Supplementary Information [file srep21400-s1.pdf]

Supporting information for:

The Influence of Structure Heights and Opening Angles of Micro- and Nanocones on the Macroscopic Surface Wetting Properties

Ling Schneider,*† Milan Laustsen,† Nikolaj Mandsberg,† and Rafael Taboryski*†

†Department of Micro- and Nanotechnology, Technical University of Denmark, 2800 Kongens Lyngby, Denmark

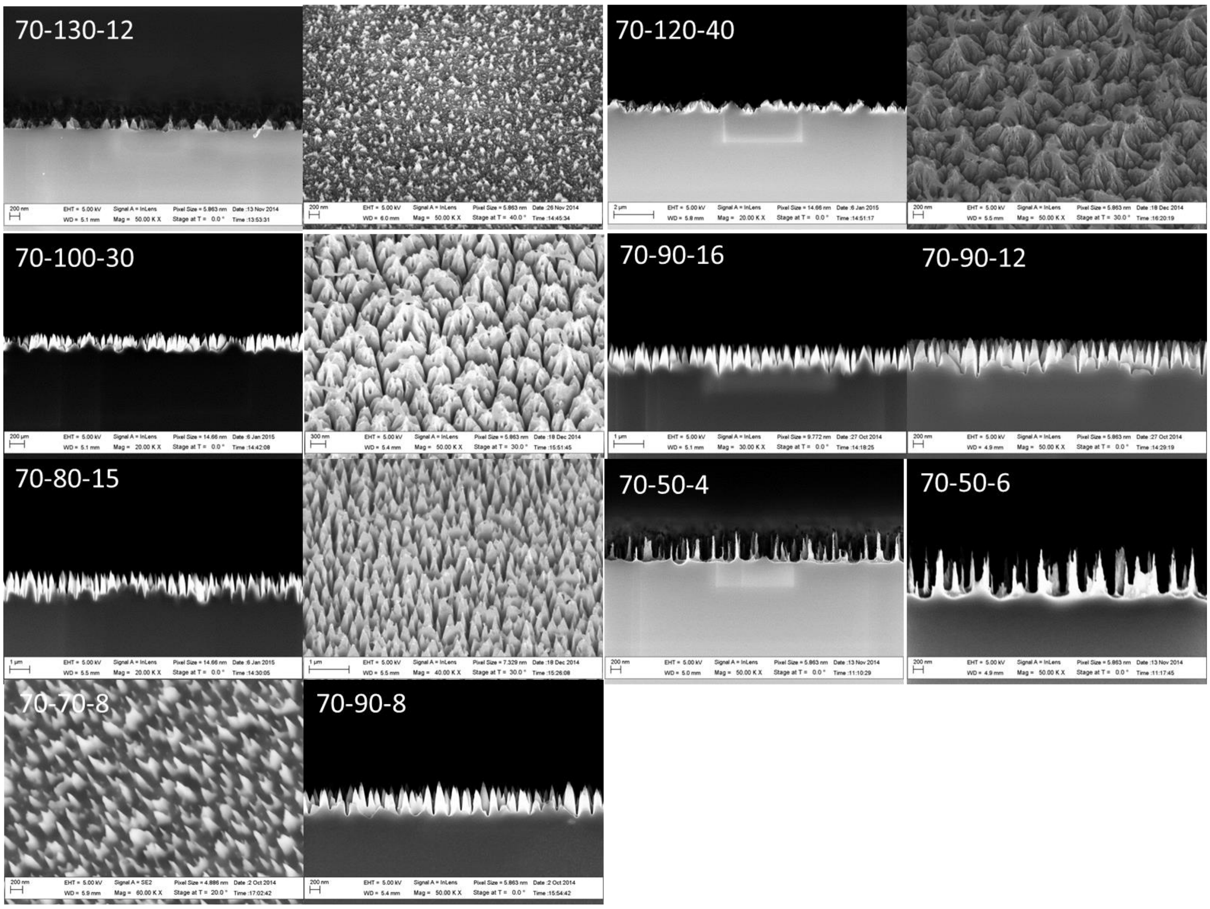

Figure S1. SEM images of Si samples prepared by RIE of different parameters.

Table S1. Summary of processing parameters, structural heights, and contact angles.

SF_6:O_2	time (min)	Height (µm)	WCA (°)	ROA (°)	θ_A (°)	θ_R
plain			113.7 ± 1.3	42.3 ± 1.3	115.5 ± 0.9	94.5 ± 2.2
70:150	8	0.040	144.1 ± 2.9	pinned	N/A	N/A
70:130	8	0.090	129.9 ± 6.0	pinned	N/A	N/A
70:130	12	0.176	158.3 ± 1.7	3 out of 5 pinned	164.8 ± 5.1	109.0 ± 2.8
70:120	40	0.616	152.3 ± 1.9	30.3 ± 2.0	157.4 ± 1.8	107.4 ± 1.4
70:110	8	0.264	133.8 ± 1.6	49.1 ± 3.6	141.8 ± 1.0	107.2 ± 3.3

70:110	40	0.733	139.1 ± 0.5	43.1 ± 5.9	152.9 ± 3.8	126.2 ± 3.0
70:100	30	0.792	146.5 ± 2.0	25.9 ± 2.3	155.1 ± 1.2	117.2 ± 4.4
70:90	8	0.557	150.2 ± 0.9	27.5 ± 0.9	159.1 ± 2.1	117.5 ± 1.3
70:90	12	0.586	140.4 ± 1.6	31.9 ± 0.4	149.2 ± 2.4	111.5 ± 3.4
70:90	16	0.821	145.7 ± 0.6	23.1 ± 0.5	154.7 ± 1.8	122.7 ± 1.2
70:80	15	1.378	152.0 ± 0.6	15.1 ± 2.2	161.6 ± 1.0	132.1 ± 2.9
70:70	8	0.733	161.4 ± 1.4	5.6 ± 2.2	163.9 ± 0.8	148.2 ± 5.4
80:80	8	0.718	156.7 ± 0.6	7.1 ± 1.1	161.6 ± 1.4	138.0 ± 1.4
80:80	12	1.070	158.2 ± 2.2	6.4 ± 0.4	162.6 ± 1.3	137.7± 1.0
70:50	4	0.557	161.3 ± 1.8	2.2 ± 0.8	164.5 ± 1.1	156.4 ± 2.9
70:50	6	0.977	165.4 ± 1.8	3.5 ± 2.3	166.1 ± 1.8	154.7 ± 5.6
70:50	8	1.540	165.5 ± 1.8	0.9 ± 0.3	167.3 ± 3.0	166.1 ± 3.3

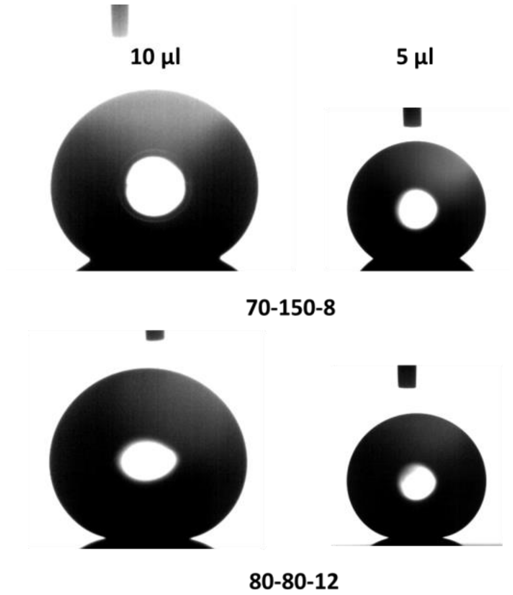

Figure S2. Images of water droplets of 10 µl and 5 µl on samples of 70-150-8 and 80-80-12. The outer size of the needle is 0.3 mm.

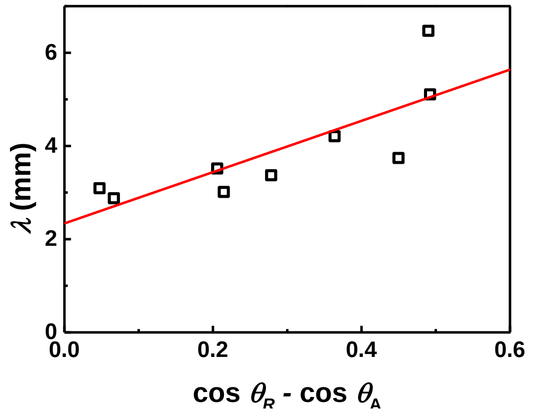

Figure S3. Effective three-phase contact line λ with respect to the contact angle hysteresis.

Measuring opening angles of individual cones

To measure the opening angles of the cones, we tried both automatic and manual methods. For the automatic method we made a Matlab script to detect the grey value of the images, while for the manual method we used the "Angle tool" in ImageJ to manually select the contour of individual cones and measure the angle between the two lines, as indicated in **Figure S4**. The automatic detection worked well with uniform cones, e.g. 70-90-8 and 70-70-8, but failed on structures of high heterogeneity. The algorithm did not work very well when the cones present a tapered contour, and tended to average the two slopes, which resulted smaller values than the manual method. Therefore, in this paper we use the manual method to measure the opening angles. To reduce the measurement error, for each sample, we measured on min. two different positions and selected min. 12 representative cones. The measurement error caused by the manual selection is unavoidable, but can be minimized by having the same person perform the data processing.

The error bars in **Figure 2b** are the reflection of two causes: the measurement error and the heterogeneity of the cones fabricated by RIE. The sharper the cones are, the more difficult the manual detection is; thus the higher the measurement error is. Such error tendency results in higher percentage of the error bar of sharper cones than blunter cones. Nevertheless, the measurement errors do not influence our discussion, as in this paper, we only discuss the relative dependence of the surface wettability.

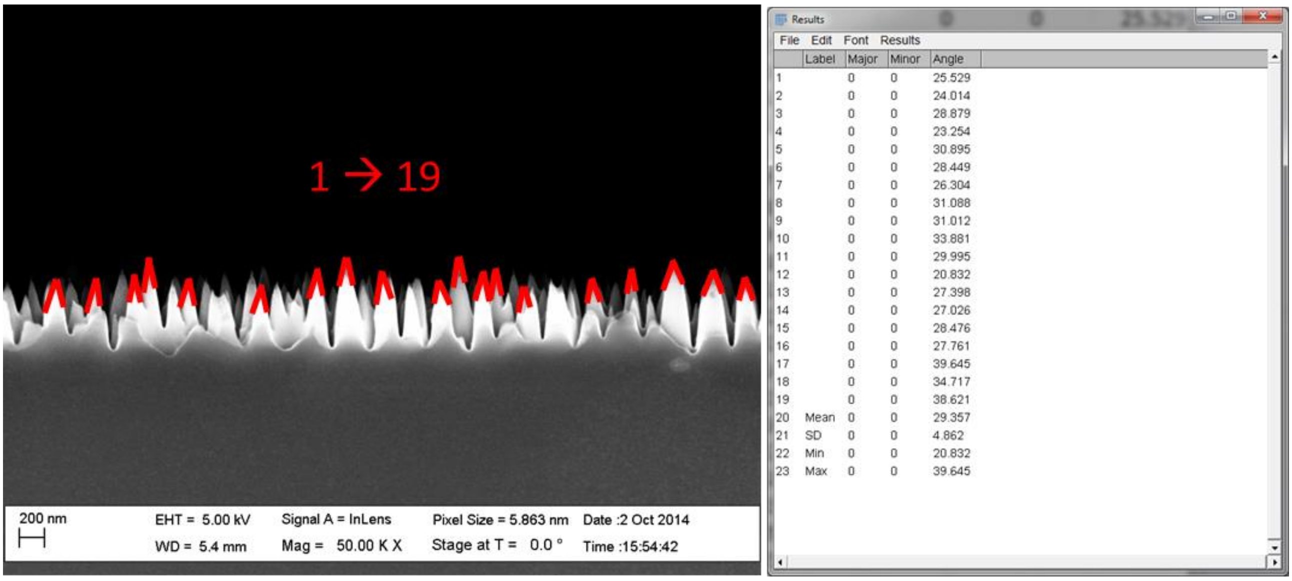

	Label	Major	Minor	Angle
1		0	0	25.529
2		0	0	24.014
3		0	0	28.879
4		0	0	23.254
5		0	0	30.895
6		0	0	28.449
7		0	0	26.304
8		0	0	31.088
9		0	0	31.012
10		0	0	33.881
11		0	0	29.995
12		0	0	20.832
13		0	0	27.398
14		0	0	27.026
15		0	0	28.476
16		0	0	27.761
17		0	0	39.645
18		0	0	34.717
19		0	0	38.621
20	Mean	0	0	29.357
21	SD	0	0	4.862
22	Min	0	0	20.832
23	Max	0	0	39.645

Figure S4. An SEM image (left) showing how opening angles of individual cones were measured and the corresponding results (right) obtained by ImageJ. The red lines are only to indicate how the contour the cones was measured.

Influence of different packing geometries on Equations 3 and 5

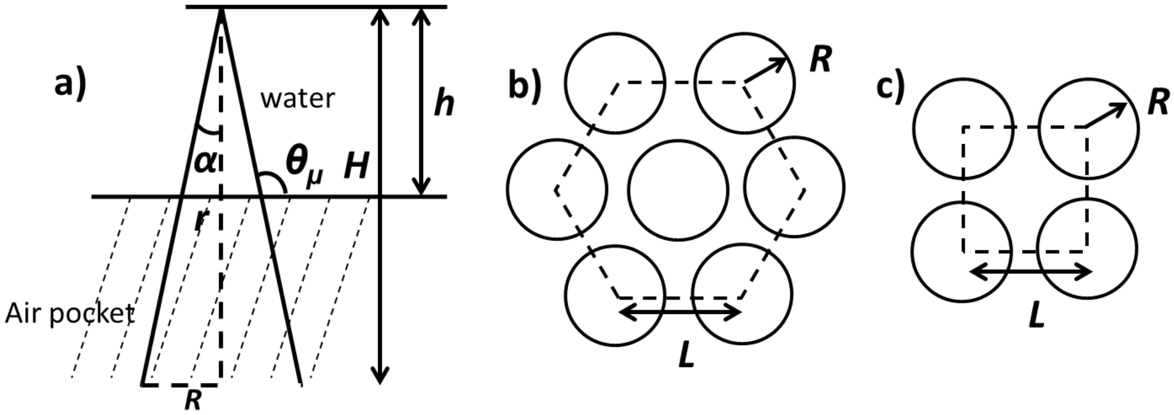

Figure S5. (a) Side view of a partially wetted cone; schematic sketches of hexagonal (b) and square (c) packed cone geometry.

The packing geometry only influences A_{lg}, while A_{ls} is independent on the packing geometry, as indicated in **Equation (2)**. Here we discuss the hexagonal and square packed geometry separately.

For hexagonal packed geometry indicated in **Figure S3b**:

$$A_{lg} = \frac{\sqrt{3}}{2}L^2 - \pi h^2 \tan^2\alpha \geq 0, \text{with } L \geq 2R. \qquad (S1)$$

Equation (1) can be rewritten as,

$$\cos\theta_e = \frac{\pi\left(\frac{h}{H}\right)^2(\cos\theta_Y + 1)}{\left(\frac{\sqrt{3}}{2}\left(\frac{L}{H}\right)^2\cot^2\alpha - \left(\frac{h}{H}\right)^2\pi\right)\sin\alpha + \pi\left(\frac{h}{H}\right)^2} - 1. \qquad \text{(S2)}$$

The effective three-phase contact length along the rolling direction,

$$\lambda = \frac{d}{L}\cdot\left(2\frac{1-\sin\alpha}{\cos\alpha}\frac{h}{L} + 1\right). \qquad \text{(S3)}$$

For the hexagonal closed packed geometry, $\mathrm{L} = 2\mathrm{R} = 2\mathrm{H}\tan\alpha$. **Equation S2** can thus be simplified to **Equation 3**; and λ in **Equation S3** is the same as presented in the main text.

For square packed geometry indicated in **Figure S3c**:

$$A_{lg} = L^2 - \pi h^2 \tan^2\alpha \geq 0, \text{with } L \geq 2R. \qquad \text{(S4)}$$

Equation (1) is thus rewritten as,

$$\cos\theta_e = \frac{\pi\left(\frac{h}{H}\right)^2(\cos\theta_Y + 1)}{\left(\left(\frac{L}{H}\right)^2\cot^2\alpha - \left(\frac{h}{H}\right)^2\pi\right)\sin\alpha + \pi\left(\frac{h}{H}\right)^2} - 1, \qquad \text{(S5)}$$

and λ remains the same as expressed in Equation S3.

Apparently the selection of different packing geometry only changes a small prefactor in **Equation (1)**, which does not alter the corresponding discussion in the main text.

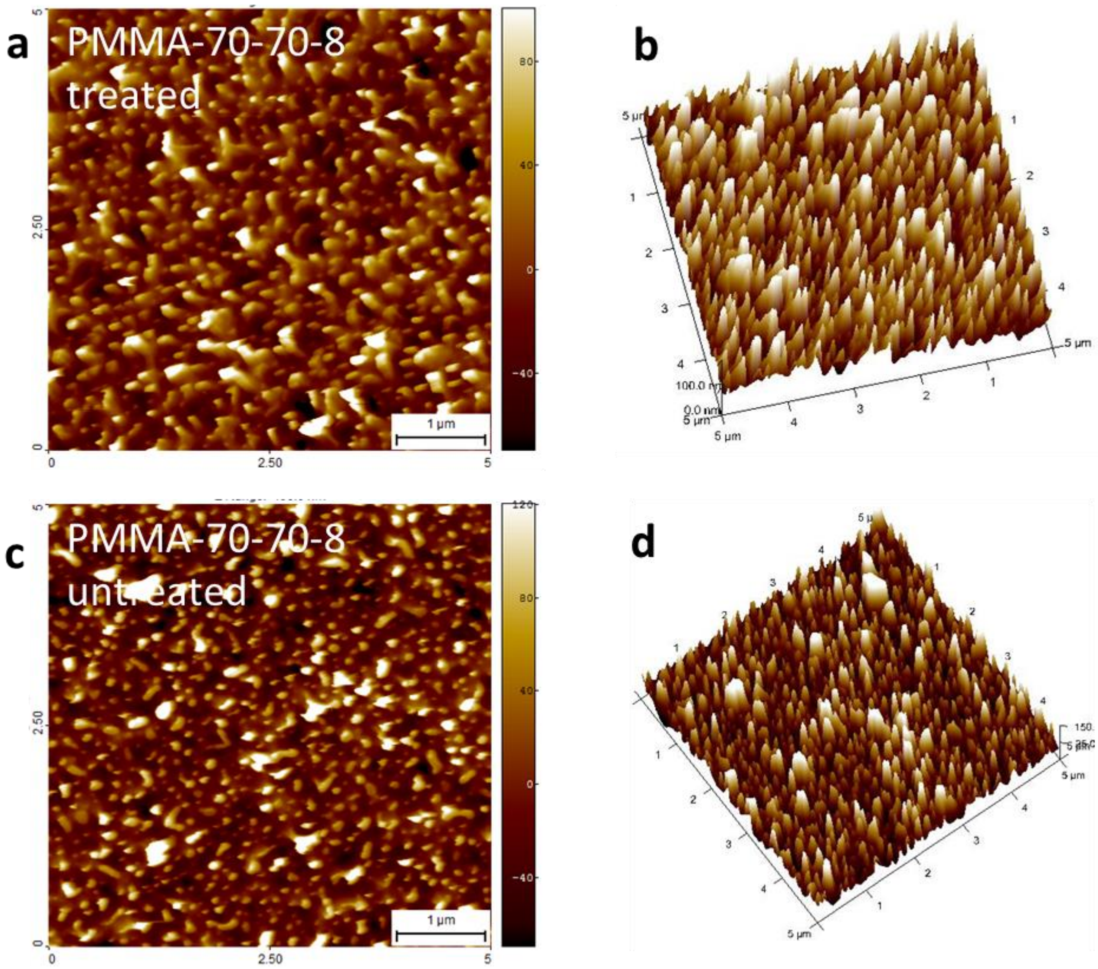

Figure S6. AFM 2D and 3D images of treated (a and b) and untreated (c and d) PMMA samples.

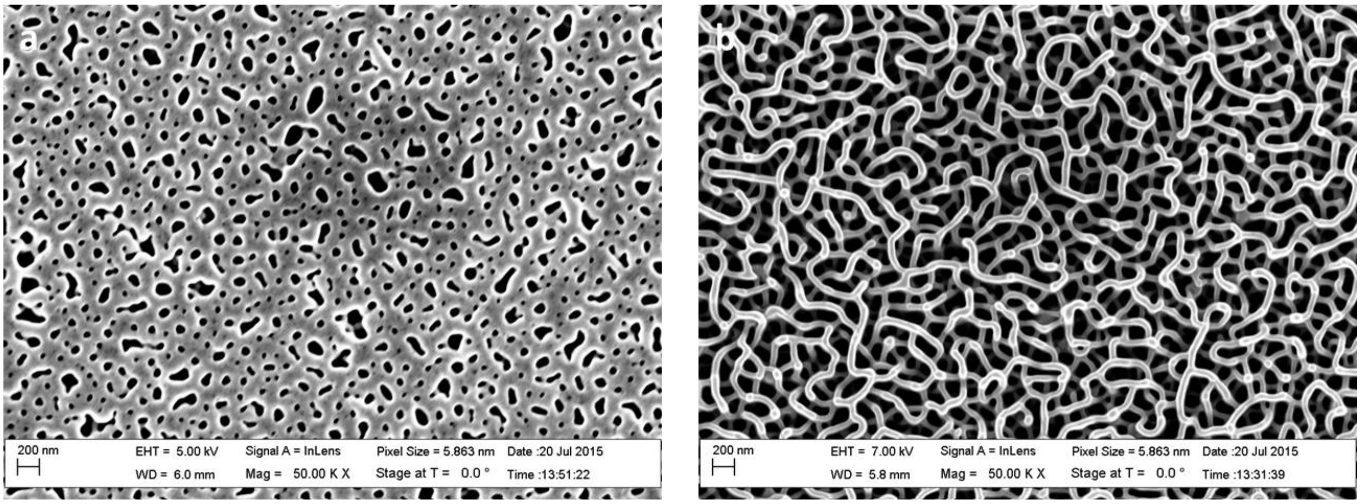

Figure S7. SEM top view images of Ni shims. a) Ni-70-70-8; b) Ni-70-90-8.

MVD Process description

The standard MVD process that was used, consists of four identical individual cycles. During each cycle, 1 injection of FDTS at 0.500 Torr and 1 injection of water at 6 Torr react for 15 minutes. The process chamber is evacuated at the end of each cycle. The total process time is around 80 min.
